# Supplementary material for: Impact of Outpatient Rehabilitation Service in Preventing the Deterioration of the Care-Needs Level Among Japanese Older Adults Availing Long-Term Care Insurance: A Propensity Score Matched Retrospective Study
Source: Int J Environ Res Public Health. 2019 Apr 10;16(7):1292. doi: 10.3390/ijerph16071292 (PMC6480242; doi:10.3390/ijerph16071292)
Supplement: Supplementary file 1 [file ijerph-16-01292-s001.pdf]

Supplementary Table S1. Degree of independent daily living for disabled older adults

Rank J	Although the patient has some disability, they are independent in their daily life and can go out on their own.
	1. The patients can go out by using public transportation.
	2. The patient can go out in the neighborhood.
Rank A	The patients can live almost independently at home, but they cannot go out without caregiver.
	1. The patient goes out with assistance and mostly stays away from the bed during the daytime.
	2. The patient seldom goes out, and they remain sleeping or waking up during the daytime.
Rank B	The patient requires some care at home and mainly stays on the bed during the daytime.
	1. The patient can transfer to the wheelchair and eat and excrete away from the bed.
	2. The patient can transfers to the wheelchair with assistance.
Rank C	The patient is bedridden and requires assistance with excretion, eating, and dressing.
	1. The patient can roll over.
	2. The patient cannot roll over.

Ministry of Health, Labor and Welfare. Textbook for expert investigators concerning a certification of needed long-term care, revised edition; Ministry of Health, Labour and Welfare: Tokyo, Japan, 2009. Available online: https://www.mhlw.go.jp/file/06-Seisakujouhou-12300000-Roukenkyoku/0000077237.pdf. [in Japanese] (Accessed on 23 March 2019).

Supplementary Table S2. Degree of independent daily living for older adults with dementia

Rank		Criteria	Examples of observed symptoms or behaviors
0		Independent	
I		The patient has some dementia, but can live almost fully independently at home and in society.	
II		Although the patient has some symptoms or behaviors disturbing their daily living, they can live independently by with the attention and support of others.	
	IIa	The condition in II is also observed outside the home.	Frequently gets lost on the streets or makes noticeable mistakes in matters that the person was previously able to handle, such as shopping, clerical work, or financial management.
	IIb	The condition in II is also observed at home.	Being unable to manage own medication regimen or stay alone at home because of the inability to respond to a call or visitor.
III		The patient has symptoms or behaviors that disturb daily living and require care.	
	IIIa	The conditions in III is mainly present during the daytime.	Has difficulty or takes extra time for dressing, eating, defecating, or urinating; puts objects into their mouth, picks up and collects objects, is incontinent, makes loud and incoherent screams, carelessly handles fire, engages in unhygienic acts or inappropriate sexual acts, etc.
	IIIb	The conditions in III is mainly present during the nighttime.	Same as rank IIIa.
IV		The patient frequently has symptoms or behaviors that disturb their daily living, and they always require care.	Same as rank III.
M		The patient has severe mental symptoms, behavioral, and psychological symptoms of dementia, or severe physical disease, and therefore requires special treatment.	Hs continued mental symptoms including delirium, delusions, and agitation and its associated problematic behaviors, such as self-mutilation or harm to others.

Ministry of Health, Labor and Welfare. Textbook for expert investigators concerning a certification of needed long-term care, revised edition; Ministry of Health, Labour and Welfare: Tokyo, Japan, 2009. Available online: https://www.mhlw.go.jp/file/06-Seisakujouhou-12300000-Roukenkyoku/0000077237.pdf. [in Japanese] (Accessed on 23 March 2019).
